# Supplementary material for: Clinical and Microbiologic Analysis of Klebsiella pneumoniae Infection: Hypermucoviscosity, Virulence Factor, Genotype, and Antimicrobial Susceptibility
Source: Diagnostics (Basel). 2024 Apr 10;14(8):792. doi: 10.3390/diagnostics14080792 (PMC11048833; doi:10.3390/diagnostics14080792)
Supplement: Supplementary file 1 [file diagnostics-14-00792-s001.zip › Supplemental_Table_1.pdf]

diagnostics

Table S1. Baseline characteristics and clinical presentations of *Klebsiella pneumoniae* isolates according to hypermucoviscosity.

	**String (–) (n = 259)**	**String (+) (n = 155)**	***p* Value**
Epidemiology			
Male sex	141 (54.4)	107 (69.0)	0.003
Age (years)	68.18 ± 13.48	67.13 ± 13.15	0.440
Underlying diseases			
Solid tumor	88 (34.0)	31 (20.0)	0.002
Chronic liver disease	27 (10.4)	23 (14.8)	0.182
Neurological disease	114 (44.0)	55 (35.5)	0.087
Chronic renal disease	29 (11.2)	10 (6.5)	0.110
Diabetes mellitus	94 (36.3)	52 (33.5)	0.572
Chronic lung disease	27 (10.4)	15 (9.7)	0.807
Solid-organ transplantation	9 (3.5)	3 (1.9)	0.547
McCabe classification			
Nonfatal underlying disease	187 (72.2)	128 (82.6)	0.017
Ultimately fatal disease	72 (27.8)	27 (17.4)	
Predisposing factors			
Urinary catheter	133 (51.4)	62 (40.0)	0.025
Percutaneous catheter drainage	44 (17.0)	44 (28.4)	0.006
L-tube	74 (28.6)	20 (12.9)	<0.001
Invasive procedure	59 (22.8)	58 (37.4)	0.001
Recent operation	55 (21.2)	18 (11.6)	0.013
Prior ICU admission within 1 month	47 (18.1)	8 (5.2)	<0.001
Category of infection			
Community-acquired infection	72 (27.8)	95 (61.3)	<0.001
Healthcare-associated infection	55 (21.2)	25 (16.1)	0.203
Nosocomial infection	132 (51.0)	35 (22.6)	<0.001
Infection source			
Urinary tract infection	72 (27.8)	16 (10.3)	<0.001
Intra-abdominal infection	57 (22.0)	57 (36.8)	0.001
Respiratory infection	100 (38.6)	63 (40.6)	0.682
Clinical presentation			
Severe sepsis and septic shock	87 (33.6)	47 (30.3)	0.492
Metastatic infection	1 (0.4)	4 (2.6)	0.068
Concomitant bacteremia	83 (32.0)	59 (38.1)	0.212
Mechanical ventilation	37 (14.3)	30 (19.4)	0.175
Admission to ICU	62 (23.9)	44 (28.4)	0.315
Treatment outcomes			
Treatment failure (72 h)	41 (15.8)	29 (18.7)	0.449
Infection-related 30-day mortality	26 (12.3)	18 (15.7)	0.400

Values are presented as n (%) or mean ± standard deviation.
ICU: intensive care unit.
