# Supplementary material for: Clinical and Microbiologic Analysis of Klebsiella pneumoniae Infection: Hypermucoviscosity, Virulence Factor, Genotype, and Antimicrobial Susceptibility
Source: Diagnostics (Basel). 2024 Apr 10;14(8):792. doi: 10.3390/diagnostics14080792 (PMC11048833; doi:10.3390/diagnostics14080792)
Supplement: Supplementary file 1 [file diagnostics-14-00792-s001.zip › Supplemental_Table_2.pdf]

diagnostics MDPI

Table S2. Antimicrobial resistance and microbiological characteristics of *Klebsiella pneumoniae* isolates according to hypermucoviscosity.

	String (–) (n = 259)	String (+) (n = 155)	*p* Value
Antimicrobial resistance rates			
Amikacin	15 (5.8)	4 (2.6)	0.131
Amoxicillin/clavulanate	109 (42.1)	14 (9.0)	<0.001
Aztreonam	122 (47.1)	15 (9.7)	<0.001
Cefazolin	124 (47.9)	16 (10.3)	<0.001
Cefepime	120 (46.3)	15 (9.7)	<0.001
Cefotaxime	121 (46.7)	16 (10.3)	<0.001
Cefoxitin	34 (13.1)	14 (9.0)	0.208
Ceftazidime	121 (46.7)	16 (10.3)	<0.001
Ciprofloxacin	98 (37.8)	13 (8.4)	<0.001
Ertapenem	0	0	n/a
Gentamicin	62 (23.9)	10 (6.5)	<0.001
Imipenem	0	0	n/a
Piperacillin/tazobactam	90 (34.7)	10 (6.5)	<0.001
Tigecycline	33 (12.7)	11 (7.1)	0.071
Trimethoprim/sulfamethoxazole	92 (35.5)	9 (5.8)	<0.001
ESBL positivity	120 (46.3)	15 (9.7)	<0.001
Serotype			
K1	16 (6.2)	61 (39.4)	<0.001
K2	15 (5.8)	44 (28.4)	<0.001
K5	2 (0.8)	1 (0.6)	0.999
K20	11 (4.2)	8 (5.2)	0.667
K54	2 (0.8)	2 (1.3)	0.632
K57	3 (1.2)	13 (8.4)	<0.001
ND	210 (81.1)	26 (16.8)	<0.001
Virulence gene			
rmpA	43 (16.6)	122 (78.7)	<0.001
magA	15 (5.8)	48 (31.0)	<0.001
allS	22 (8.6)	58 (37.4)	<0.001
mrkD	252 (98.1)	152 (98.1)	0.999
entB	253 (98.4)	155 (100.0)	0.302
kfu	71 (27.6)	67 (43.2)	0.001
aerobactin	48 (18.5)	122 (78.7)	<0.001
String test			
Biofilm mass	0.68 ± 0.40	0.72 ± 0.64	0.521

Values are presented as n (%) or mean ± standard deviation.
n/a: not available; ESBL: extended-spectrum β-lactamase; ND: not detected.
