# Supplementary material for: Clinical and Microbiologic Analysis of Klebsiella pneumoniae Infection: Hypermucoviscosity, Virulence Factor, Genotype, and Antimicrobial Susceptibility
Source: Diagnostics (Basel). 2024 Apr 10;14(8):792. doi: 10.3390/diagnostics14080792 (PMC11048833; doi:10.3390/diagnostics14080792)
Supplement: Supplementary file 1 [file diagnostics-14-00792-s001.zip › Supplemental_Table_3.pdf]

Table S3. Baseline characteristics and clinical presentations of *Klebsiella pneumoniae* isolates according to aerobactin positivity.

	**Aerobactin (–) (n = 244)**	**Aerobactin (+) (n = 170)**	***p* Value**
Epidemiology			
Male sex	134 (54.9)	114 (67.1)	0.013*
Age (years)	67.55 ± 13.68	68.12 ± 12.90	0.673
Underlying diseases			
Solid tumor	87 (35.7)	32 (18.8)	<0.001
Chronic liver disease	29 (11.9)	21 (12.4)	0.886
Neurological disease	102 (41.8)	67 (39.4)	0.626
Chronic renal disease	29 (11.9)	10 (5.9)	0.040
Diabetes mellitus	80 (32.8)	66 (38.8)	0.206
Chronic lung disease	26 (10.7)	16 (9.4)	0.680
Solid-organ transplantation	11 (4.5)	1 (0.6)	0.018*
McCabe classification			
Nonfatal underlying disease	174 (71.3)	141 (82.9)	0.006
Ultimately fatal disease	70 (28.7)	29 (17.1)	
Predisposing factors			
Urinary catheter	122 (50.0)	73 (42.9)	0.157
Percutaneous catheter drainage	43 (17.6)	45 (26.5)	0.030
L-tube	68 (27.9)	26 (15.3)	0.003
Invasive procedure	58 (23.8)	59 (34.7)	0.015
Recent operation	55 (22.5)	18 (10.6)	0.002
Prior ICU admission within 1 month	40 (16.4)	15 (8.8)	0.026
Category of infection			
Community-acquired infection	69 (28.3)	98 (57.6)	<0.001
Healthcare-associated infection	52 (21.3)	28 (16.5)	0.220
Nosocomial infection	123 (50.4)	44 (25.9)	<0.001
Infection source			
Urinary tract infection	69 (28.3)	19 (11.2)	<0.001
Intra-abdominal infection	57 (23.4)	57 (33.5)	0.023
Respiratory infection	88 (36.1)	75 (44.1)	0.099
Clinical presentation			
Severe sepsis and septic shock	85 (34.8)	49 (28.8)	0.198
Metastatic infection	1 (0.4)	4 (2.4)	0.164
Concomitant bacteremia	85 (34.8)	57 (33.5)	0.783
Mechanical ventilation	36 (14.8)	31 (18.2)	0.344
Admission to ICU	55 (22.5)	51 (30.0)	0.087
Treatment outcomes			
Treatment failure (72 h)	38 (15.6)	32 (18.8)	0.385
Infection-related 30-day mortality	25 (12.4)	19 (15.3)	0.450
Acute kidney injury	32 (13.1)	19 (11.2)	0.555

Values are presented as n (%) or mean ± standard deviation.
*Fisher's exact test.
ICU: intensive care unit.
