# Supplementary material for: Clinical and Microbiologic Analysis of Klebsiella pneumoniae Infection: Hypermucoviscosity, Virulence Factor, Genotype, and Antimicrobial Susceptibility
Source: Diagnostics (Basel). 2024 Apr 10;14(8):792. doi: 10.3390/diagnostics14080792 (PMC11048833; doi:10.3390/diagnostics14080792)
Supplement: Supplementary file 1 [file diagnostics-14-00792-s001.zip › Supplemental_Table_4.pdf]

diagnostics

Table S4. Antimicrobial resistance and microbiological characteristics of *Klebsiella pneumoniae* isolates according to aerobactin positivity.

	Aerobactin (–) (n = 244)	Aerobactin (+) (n = 170)	*p* Value
Antimicrobial resistance rates			
Amikacin	12 (4.9)	7 (4.1)	0.702
Amoxicillin/clavulanate	100 (41.0)	23 (13.5)	<0.001
Aztreonam	113 (46.3)	24 (14.1)	<0.001
Cefazolin	116 (47.5)	24 (14.1)	<0.001
Cefepime	111 (45.5)	24 (14.1)	<0.001
Cefotaxime	113 (46.3)	24 (14.1)	<0.001
Cefoxitin	30 (12.3)	18 (10.6)	0.594
Ceftazidime	113 (46.3)	24 (14.1)	<0.001
Ciprofloxacin	94 (38.5)	17 (10.0)	<0.001
Ertapenem	0	0	n/a
Gentamicin	56 (23.0)	16 (9.4)	<0.001
Imipenem	0	0	n/a
Piperacillin/tazobactam	80 (32.8)	20 (11.8)	<0.001
Tigecycline	33 (13.5)	11 (6.5)	0.022
Trimethoprim/sulfamethoxazole	97 (39.8)	4 (2.4)	<0.001
ESBL positivity	111 (45.5)	24 (14.1)	<0.001
Serotype			
K1	11 (4.5)	66 (38.8)	<0.001
K2	16 (6.6)	43 (25.3)	<0.001
K5	2 (0.8)	1 (0.6)	0.999
K20	6 (2.5)	13 (7.6)	0.013
K54	3 (1.2)	1 (0.6)	0.647
K57	2 (0.8)	14 (8.2)	<0.001
ND	204 (83.6)	32 (18.8)	<0.001
Virulence gene			
rmpA	6 (2.5)	159 (93.5)	<0.001
magA	3 (1.2)	60 (35.3)	<0.001
allS	8 (3.3)	72 (42.4)	<0.001
mrkD	237 (97.9)	167 (98.2)	0.999*
entB	239 (98.8)	169 (99.4)	0.646*
kfu	60 (24.8)	78 (45.9)	<0.001
aerobactin			
String test	33 (13.5)	122 (71.8)	<0.001
Biofilm mass	0.65 ± 0.36	0.76 ± 0.65	0.059

Values are presented as n (%) or mean ± standard deviation.
*Fisher's exact test.
n/a: not available; ESBL: extended-spectrum β-lactamase; ND: not detected.
